# HSP70 Inhibition Leads to the Activation of Proteasomal System under Mild Hyperthermia Conditions in Young and Senescent Fibroblasts

**DOI:** 10.1155/2020/9369524

**Published:** 2020-02-27

**Authors:** Perinur Bozaykut, Erdi Sozen, Elif Kaga, Asli Ece, Esra Ozaltin, Jonas Bergquist, Nesrin Kartal Ozer, Betul Karademir Yilmaz

**Affiliations:** ^1^Department of Biochemistry, School of Medicine, Marmara University, 34854 Istanbul, Turkey; ^2^Department of Molecular Biology and Genetics, Acibadem Mehmet Ali Aydinlar University, 34752 Istanbul, Turkey; ^3^Genetic and Metabolic Diseases Research and Investigation Center, Marmara University, 34854 Istanbul, Turkey; ^4^Health Application and Research Center, University of Health Sciences, Afyonkarahisar, Turkey; ^5^Department of Chemistry-BMC, Analytical Chemistry, Uppsala University, Uppsala, Sweden

## Abstract

Aging has been characterized with the accumulation of oxidized proteins, as a consequence of progressive decline in proteostasis capacity. Among others, proteasomal system is an efficient protein turnover complex to avoid aggregation of oxidized proteins. Heat shock protein 70 (HSP70) is another critical player that is involved in some key processes including the correct folding of misfolded proteins and targeting aggregated proteins to the proteasome for rapid degradation. The aim of this study was to determine the role of proteasomal system and heat shock proteins to maintain proteome balance during replicative senescence in mild hyperthermia conditions. Our results demonstrated that HSP40/70 machinery is induced by mild hyperthermia conditions independent from senescence conditions. Since HSP70 is largely responsible for the rapidly inducible cell protection following hyperthermia, the activation of “heat shock response” resulted in the elevation of HSP40/70 expressions as well as the proteasome activity. Interestingly, when HSP70 expression was inhibited, increased proteasomal activation was shown to be responsive to mild hyperthermia. Since HSP70 is involved in various stress-related pathways such as oxidative and endoplasmic reticulum stress, depletion of HSP70 expression may induce proteasomal degradation to maintain proteome balance of the cell. Thus, our data suggests that in mild heat stress conditions, molecular chaperone HSP70 plays an important role to avoid protein oxidation and aggregation; however, activities of proteasomal system are induced when HSP70 expression is depleted.

## 1. Introduction

Aging is characterized by the loss of cellular function that results in the accumulation of oxidative damage to macromolecules such as lipids, DNA, and proteins [1]. Specifically, decreased protein turnover has been proposed as one of the major hallmarks of aging [1, 2]. Proteostasis state of the organisms is maintained by the balanced activity of protein synthesis, folding, and degradation network [2, 3]. However, in senescent organisms, oxidized proteins are accumulated due to the reduced efficiency of heat shock proteins (HSPs) and decline in the activity of degradation mechanisms such as proteasomal system [4, 5].

HSPs, which act as molecular chaperones, are known to have key roles in the maintenance of the proteostasis that work coordinately for the correct folding of misfolded proteins [6]. Among the members of the heat shock proteins, recent studies have pointed to 70 kDa heat shock protein (HSP70) family in protein homeostasis, since it is involved in the assembly of newly synthesized proteins and refolding of misfolded proteins, as well as targeting aggregated proteins to the proteasome for rapid degradation [7]. Exciting new data also reveals HSP70 as a regulator of proteasomal degradation, and the relationship of HSP70 and the proteasomal degradation under cellular stress conditions would explore the proteome balance in aging process [8].

A number of stress conditions lead to the activation of a highly regulated and rapid series of events, which is named as the “heat shock response” [9], and HSPs are shown to mainly involve in the response against mild heat shock [10]. HSP70 expression has been widely accepted as a marker for heat stress, and the protective role of HSP70 under hyperthermic conditions has been revealed [11–13]. However, to our knowledge, a possible function of HSP70 in the modulation of the proteasome activity against hyperthermia has not been investigated. In the current study, we are able to demonstrate the role of HSP70 on the proteasomal system not only under hypertermic conditions but also under replicative senescence conditions. Our results revealed that in mild hyperthermia, HSP70 increased to cope with the heat stress; however, when HSP70 is inhibited, the proteasomal degradation is activated to maintain the proteome balance.

## 2. Materials and Methods

### 2.1. Cell Culture and Treatment of Mild Hyperthermia

Human primary fibroblasts, isolated from the foreskin as explained previously [14], were cultured in Dulbecco's modified Eagle's medium (DMEM) containing penicillin 100 U/ml, streptomycin 100 *μ*g/ml, and 10% fetal bovine serum (FBS) in a humidified atmosphere of 5% CO_2_ and 95% air at 37°C. 2 × 10^5^ cells/ml initially were plated and then incubated for one week for young cells (passage number: 18 and population doubling (PD): 25) and three weeks for senescent cells (passage number: 33 and PD: 58) to a density of 3 × 10^6^ cells/ml. Groups are respresented as PD:25A and PD:58A for young and senescent controls, as PD:25B and PD:58B for heat stressed young and senescent cells without recovery, and as PD:25C and PD:58C for heat stressed young and senescent cells, respectively, with recovery at 37°C for 3 h throughout the texts and the figures. For mild hyperthermic conditions, cells were incubated at 42°C for 1 h in a culture medium and used immediately or used after recovery at 37°C for 3 h for the analyses.

### 2.2. Quantitative RT-PCR for the Determination of HSP mRNA Expressions

After treatment of fibroblast cells with hyperthermia, total RNA was isolated by RNeasy Mini Kit (Qiagen) as described in the manufacturer's protocol. 100 ng total RNA was used to obtain cDNA by using the iScript cDNA Synthesis kit (Biorad). Specific genes were amplified by using QuantiTect PCR Sybr Green kit (Qiagen) and Rotor Gene QRT-PCR system (Qiagen). The results were normalized to GAPDH mRNA expressions. The sequences of primers used were as follows: human HSP40 forward: TCCCAGACCCTGTACACTCC; human HSP40 reverse: TTGCTGGAGTCACTCACTGG; human HSP70 forward: AGCCAAGAAGGCAAAAGTGA; human HSP70 reverse: CCACTGCGTTCTTAGCATCA; human GAPDH forward: GATTTGGTCGTATTGGGCGC; and human GAPDH reverse: TTCCCGTTCTCAGCCTTGAC.

### 2.3. Proteasomal Activity Analysis by Fluorimetry

The proteasome activity was measured in whole cell extracts following mild hyperthermia treatment. Peptidase activity towards the fluorogenic peptide substrate succinyl–leucine–leucine–valine–tyrosine–methylcoumarin (suc-LLVY-MCA) was measured by incubation of samples for 30 min at 37°C in the reaction buffer (150 mM Tris, 30 mM KCl, 5 mM MgOAc, 5 mM MgCl_2_, 0.5 mM DTT, and 200 *μ*M suc-LLVY-MCA). For all measurements, 5 mM ATP was added to the reaction mixtures to detect both ATP and ubiquitin-dependent and -independent proteolysis. Fluorescence of the liberated methylcoumarin (MCA) was determined at 360 nm excitation and 460 nm emission wavelengths. Free MCA standards were used for calibration curves, and the amount of substrate degradation was calculated as nmol MCA/mg proteinxmin.

### 2.4. Silencing and Inhibition of HSP70

Fibroblast cells were transiently transfected with siRNA targeting to HSP70 (target siRNA), GAPDH (positive control), and nontargeting siRNA (negative control) by Accell siRNA delivery media (Dharmacon) according to the manufacturer's protocol. In summary, 2 × 10^5^ PD25 initial cells were plated and incubated at 37°C with 5% CO_2_ overnight. 25 nM final concentration of siRNA solution in 1x siRNA buffer was added to the wells, and the cells were incubated at 37°C with 5% CO_2_. Following 24 h of incubation, transfection medium was changed to complete medium for additional 48 h of incubation. After 72 h of transfection, the cells were treated by hyperthermia in the delivery media and used immediately or following culturing in a humidified atmosphere of 5% CO_2_ and 95% air at 37°C for 3 h prior to analyses. For HSP70 inhibition, 50 *μ*M of KNK437 (*N*-formyl-3,4-methylenedioxy-benzylidene-*γ*-butyrolactam) was dissolved in DMSO, and the final concentration of DMSO in each culture medium was 0.25% (*v*/*v*). The same concentration of DMSO was used in the control groups. Following 12 h of incubation, the cells were treated with mild hyperthermia as indicated above.

### 2.5. Two-Dimensional Electrophoresis following Mild Hyperthermia Treatment

Human primary fibroblasts were lysed in cell lysis buffer (100 mM Pipes, 1 mM MgCl_2_, 2 mM EDTA, 0.5% Nonidet P-40, 1 mM DTT, protease inhibitor). The protein concentrations of cell lysates were determined using colorimetric assay. For the first dimension step, 50 *μ*g of total protein was loaded onto immobilized pH gradient strips (11 cm, pH 4-7) (BioRad, USA) via passive rehydration. IPG strips were then subjected to isoelectric focusing on a Protean isoelectric focusing cell (BioRad, USA). For the second dimension, IPG strips were applied onto SDS-polyacrylamide gels. Gel imaging and spot analysis were performed using Bio 2D-Software (Vilber Lourmat).

### 2.6. Mass Spectrometric Analysis by MALDI-TOF

In-gel digestion of the proteins was performed with trypsin following standard protocols [15]. The digests were mixed with 0.6 *μ*l 1 : 1 (*v*/*v*) saturated matrix solution of *α*-cyano-4-hydroxycinnamic acid in 30% acetonitrile/0.1% trifluoroacetic acid. The mixture was spotted onto the matrix-assisted laser desorption/ionisation target plate (ground steel: 24 × 16 spots) and left to dry. The plate was inserted into the BrukerUltraflex II MALDI-TOF mass spectrometer for protein analysis. Spectra were analyzed by using mMass [16] and manual PMF searches in Mascot. Database searching was performed by at the 60-100 ppm error level using “human” and SwissProt. One missed cleavage was allowed.

### 2.7. Statistical Analyses

All statistical analyses were performed using the Prism 7 software (Graph-Pad, CA, USA). Statistical significances of differences were evaluated by one-way ANOVA test followed by multiple comparisons using the Student–Newman–Keuls test multiple comparison test. *p* < 0.05 was established as statistically significant.

## 3. Results and Discussion

### 3.1. Effects of Heat Stress on HSP40 and HSP70 mRNA Levels

The accumulation of damage to cellular macromolecules has been proposed to lead to the continuous and irreversible aging process. Recent studies indicated that elevated protein damage during aging is likely to be aggravated by the decrease in heat shock response and HSP levels and, as a result, by the deterioration of protein quality control [17]. In the present study, we analyzed the effects of mild hyperthermia on protein quality system by measuring the expressions of HSPs in young and senescent cells. The consequences of the replicative senescence by serial passaging and hyperthermia at 42°C for 1 h (without and with recovery at 37°C for 3 h) on the morphology of the fibroblast cells was evaluated by light microscopy, and the microscopic analysis showed significant change on the morphology of the senescent cells. However, we detected only a slight change in the mild hyperthermia treated cells (Supplementary [Supplementary-material supplementary-material-1]). Viability of the cells was also tested by MTT, and no significant change was found either in the replicative senescence or in the hyperthermia conditions (Supplementary [Supplementary-material supplementary-material-1]).

**Figure 1:**
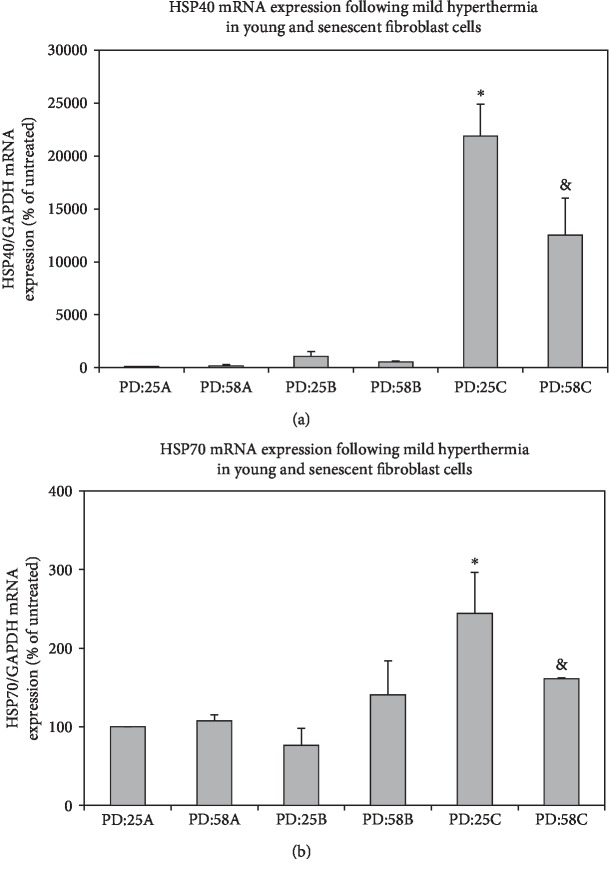
Effects of mild hyperthermia and recovery on the mRNA levels of HSPs in young and senescent fibroblast cells. Cells were treated with mild hyperthermia at 42°C for 1 h and used immediately for analysis or cultured at 37°C for 3 h as described in Materials and Methods. mRNA levels of HSP40 (a) and HSP70 (b) were measured by RT-PCR and normalized to GAPDH. Data are expressed as mean ± SD.^∗^*p* < 0.05 vs. PD:25A. ^&^*p* < 0.05 vs. PD:25C (*n* = 3).

Among other HSPs, HSP70 plays a pivotal role in cellular repair and protection against stress conditions. HSP40 and HSP70 are known to work in cohort; HSP40 is responsible for recognizing/binding unfolded proteins and transferring them to HSP70 [18]. Interestingly, we observed no significant change in both HSP40 and HSP70 mRNA expressions in senescent PD:58A cells when compared to young PD:28A cells (Figure 1). On the other hand, mRNA levels of both HSP40 and HSP70 were found to be increased following 3 h of mild hyperthermia treatment in both young and senescent cells when compared to their controls (Figure 1). Our results showed that HSP40/HSP70 chaperone machinery is induced by mild hyperthermia in either the presence or the absence of senescence conditions. Since HSP70 is rapidly induced under stress conditions [19, 20], it is likely that mild hyperthermia leads to the oxidative damage to proteins and thus, their unfolding [21, 22]. As a consequence, the activation of “heat shock response” is regulated by the elevation of HSP40/70 expressions. In contrast, in our previous study, we showed that protein expressions of HSP40 and HSP70 were increased only in young cells and there was no change in senescent cells following mild hyperhtermia [23]. This difference suggests that heat shock response that is regulated by HSP40/HSP70 machinery was induced only in mRNA levels in senescent cells.

### 3.2. Effect of HSP70 Silencing/Inhibition on the Proteostasis of Young and Senescent Cells

**Figure 2:**
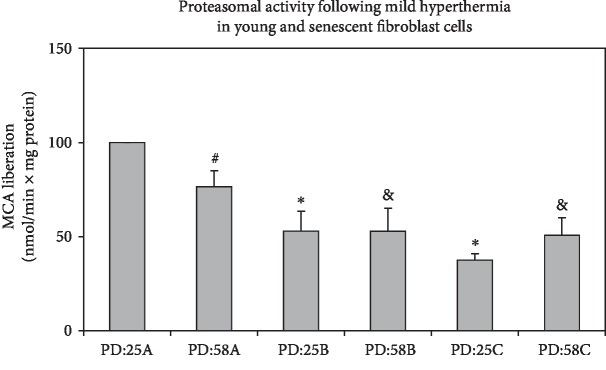
Effects of mild hyperthermia on proteasome activity in young and senescent fibroblast cells. Cells were treated as described in Materials and Methods, and proteasome activity was measured in ATP-stimulated conditions. MCA liberation was measured for 30 min, and data were normalized to min and mg protein. Data are expressed as mean ± SD. ^#,∗^*p* < 0.05 vs. PD:25A. ^&^*p* < 0.05 vs. PD:58A (*n* = 3).

It is widely known that the activity of proteasomal system is impaired during aging process [24]. Consistent with earlier studies, we observed the decline of proteasome activity in senescent fibroblasts (PD:58A) when compared to young fibroblasts (Figure 2). However, mild hyperthermia resulted in even lower proteasomal activity both in young and senescent cells when compared to their controls, PD:28A and PD:58A, respectively (Figure 2). These results implicated that in mild heat stress conditions, HSP40/70 expressions are in correlation with decreased proteasome activity.

**Figure 3:**
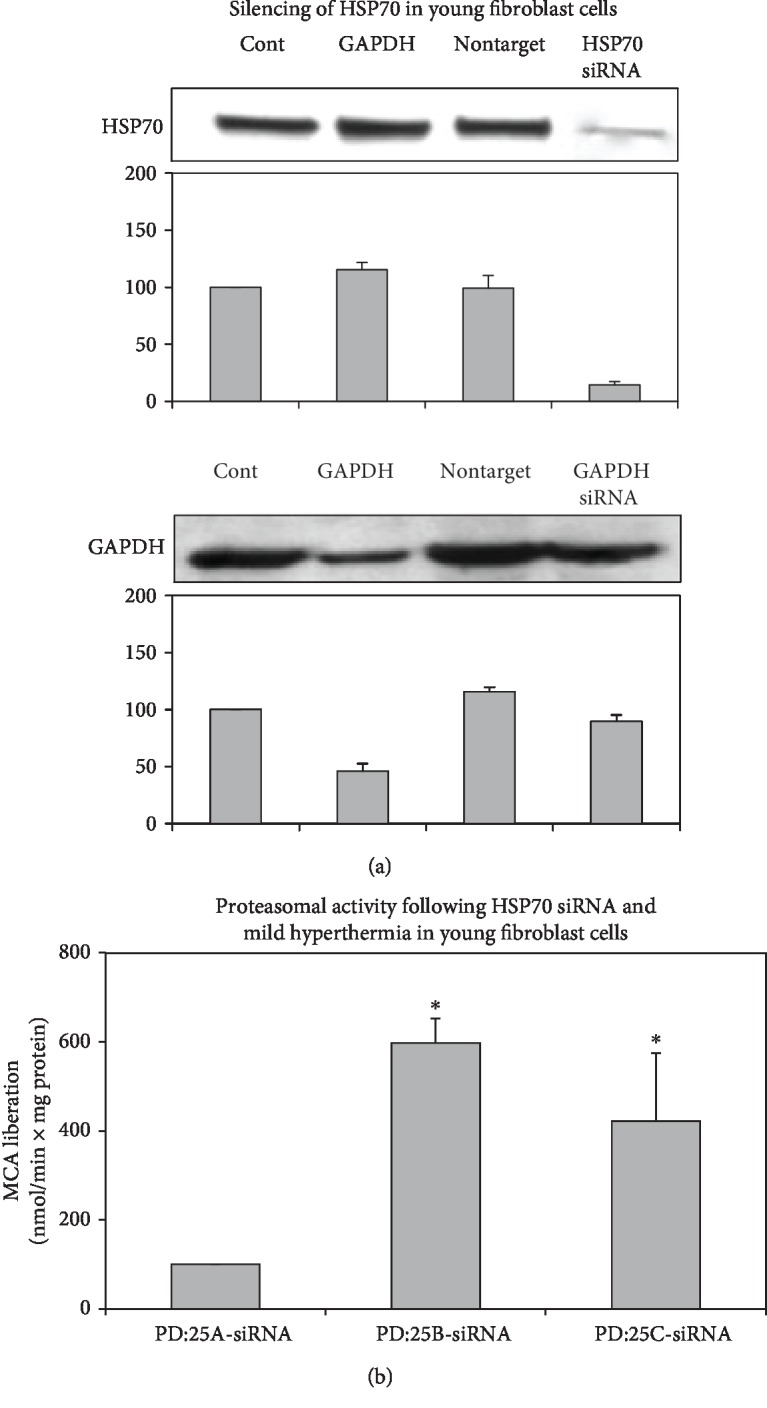
HSP70 silencing and proteasome activity in young fibroblast cells. Cells were either transfected with HSP70 or GAPDH siRNA, and the efficiency of the transfection was measured by immunoblotting. (a) Immunoblots of HSP70 and GAPDH siRNA-transfected cells (b) proteasomal activity following HSP70 siRNA and heat treatment in young fibroblast cells. Data are expressed as mean ± SD. ^∗^*p* < 0.05 vs. PD:25A-siRNA.

**Figure 4:**
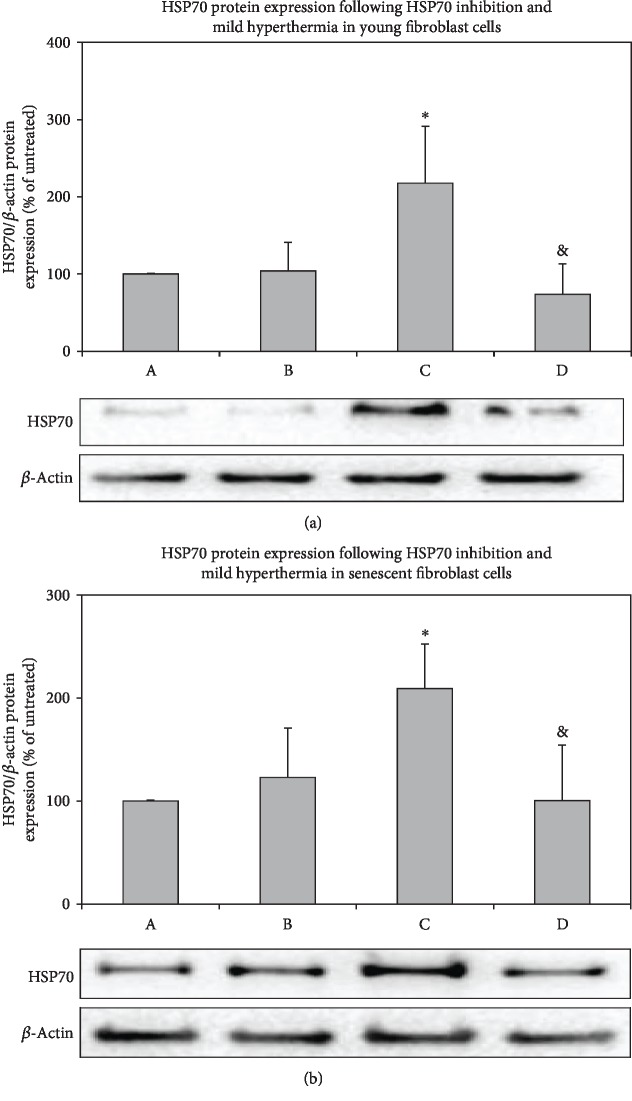
Effects of KNK437 treatment on the protein levels of HSP70 in young and senescent fibroblast cells. After 12 hours of incubation with KNK437, cells were treated with mild hyperthermia at 42°C for 1 h and cultured at 37°C for 3 h. Protein levels were measured by western blotting followed by densitometric analysis of protein bands and relative ratios were normalized to *β*-actin levels in young (a) and senescent (b) fibroblast cells. A: DMSO control, B: KNK437 control, C: DMSO+42°C for 1 h with recovery at 37°C for 3 h, and D: KNK437+42°C for 1 h with recovery at 37°C for 3 h. Data are expressed as mean ± SD. ^∗^*p* < 0.05 vs. A; ^&^*p* < 0.05 vs. C (*n* = 3).

Since our results pointed the pivotal role of HSP70 in hyperthermia so far, HSP70 protein was silenced in young fibroblasts (Figure 3) or was inhibited by KNK437 treatment in young and senescent fibroblasts (Figure 4) to reveal its further effects on the proteostasis. First, we analyzed the effect of HSP70 silencing on proteasome activity in young fibroblasts. Our results demonstrated that HSP70 silencing led to the increased proteasomal activity in response to mild hyperthermia in young cells (Figure 3(b)) indicating the possible role of proteasomal system in the protein homeostasis when HSP70 is absent. Here, we faced a limitation to transfect senescent cells by siRNA since senescent cells did not resist the HSP70 siRNA transfection and survive in enough numbers for the heat treatment.

**Figure 5:**
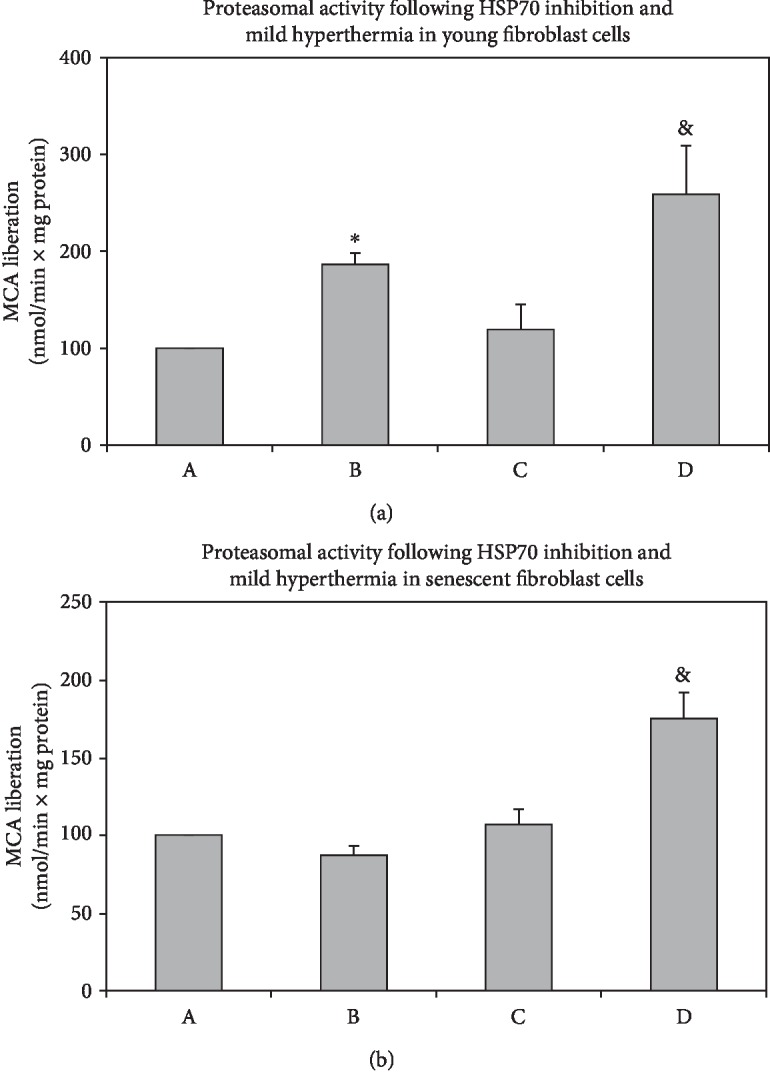
Effects of HSP70 inhibition on proteasome activity in young and senescent fibroblast cells. After 12 hours of incubation with KNK437, cells were treated with mild hyperthermia at 42°C for 1 h and cultured at 37°C for 3 h. Proteasomal activity was measured in young (a) and senescent (b) fibroblast cells. A: DMSO control, B: KNK437 control, C: DMSO+42°C for 1 h with recovery at 37°C for 3 h, and D: KNK437+42°C for 1 h with recovery at 37°C for 3 h. Data are expressed as mean ± SD. ^∗^*p* < 0.05 vs. A; ^&^*p* < 0.05 vs. C (*n* = 3).

In addition to silencing, we inhibited HSP70 induction by KNK437 pretreatment in both young and senescent cells (Figures 4(a) and 4(b)) (A: DMSO control; B: KNK437 control; C: DMSO+42°C for 1 h with recovery at 37°C for 3 h; D: KNK437+42°C for 1 h with recovery at 37°C for 3 h). In mammalian cells, the inducible isoform of HSP70 (HSP72) is highly expressed upon exposure to stressful conditions such as hyperthermia [22]. Our results showed the role of rapidly inducible cell protection by HSP72 isoform only under hyperthermia conditions. On the other hand, heat stress followed by the inhibition of HSP70 resulted in the elevation of the proteasomal activity in both young and senescent cells compared to their controls (Figures 5(a) and 5(b) and D compared with B). Since HSP70 is involved in various stress-related pathways such as oxidative and endoplasmic reticulum stress [22], the depletion of HSP70 expression may induce proteasomal degradation to maintain proteome balance of the cell. Our results showed that, as a result of increased protein oxidation and aggregation during mild hyperthermia, activities of proteasomal system increased when HSP70 expression was knockdown and inhibited.

Altered protein turnover and increased protein aggregation are two of the key markers in aging process [14]. Consistent with that data, we showed that in senescent cells (PD:58A), the proteolysis and the soluble protein levels decreased; however, protein aggregates increased when compared to young cells (Supplementary [Supplementary-material supplementary-material-1]). We also tested the effect of mild hyperthermia on protein turnover in young cells and our results showed both soluble proteins and protein aggregates decreased by mild hyperthermia when compared to their controls (Supplementary [Supplementary-material supplementary-material-1]). Following the silencing of HSP70 in young fibroblasts, overall proteolysis did not change while soluble proteins and protein aggregates decreased. When HSP70 was absent, immediate response following hyperthermia was not very significant (compare PD:25B to PD25B:siRNA in Supplementary Figures [Supplementary-material supplementary-material-1]).

### 3.3. 2D Gel Electrophoresis and MALDI-TOF MS Analysis to Determine the Differentially Expressed Proteins in Young and Senescent Fibroblasts following Mild Hyperthermia

**Figure 6:**
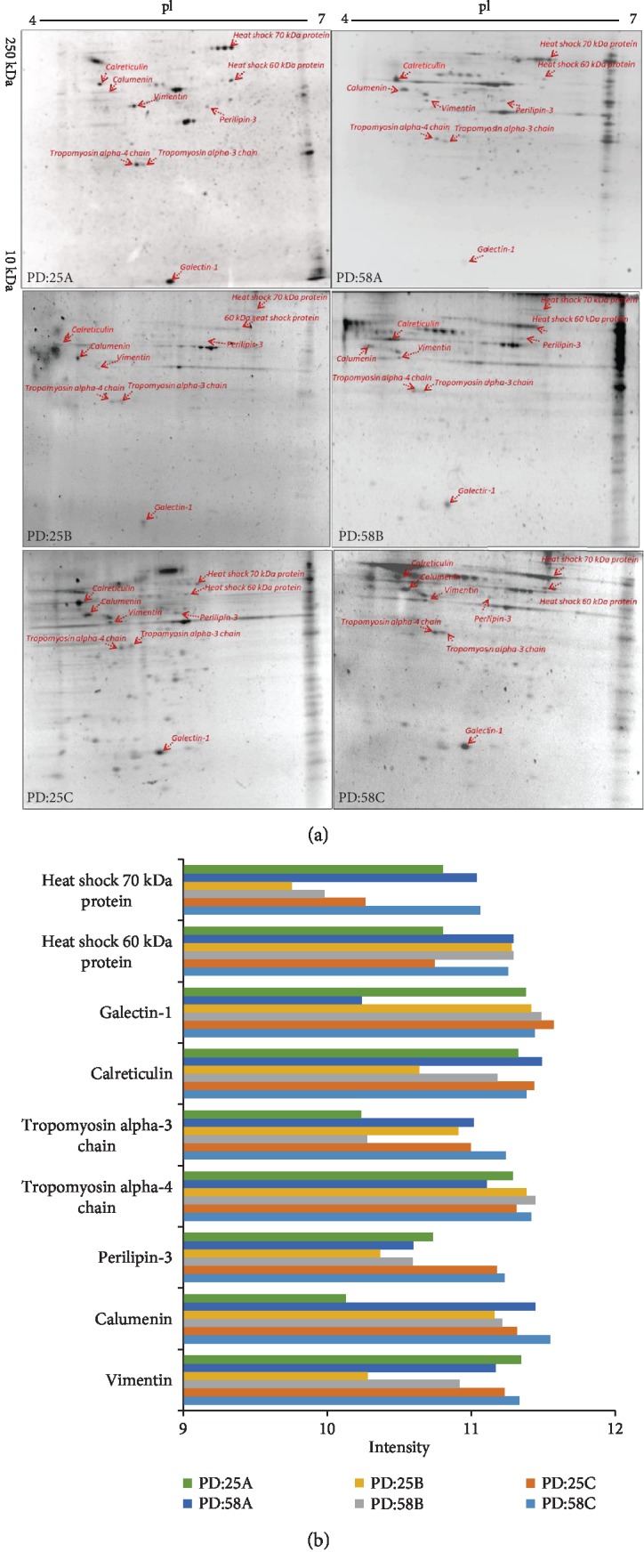
2D gel images of proteins displaying differential levels in young and senescent fibroblast cells following heat stress. Cells were treated with mild hyperthermia at 42°C for 1 h and used immediately for analysis or cultured at 37°C for 3 h. Cells were harvested and protein spots were determined by two-dimensional gel electrophoresis (a) followed by identification of protein bands via MALDI-TOF MS (b).

To investigate the differences in protein expressions in young and senescent fibroblasts treated with hyperthermia, we performed gel-based proteomics, and the protein spots obtained from 2D gels were analyzed by MALDI-TOF MS. Proteins identified by mass spectrometer (heat shock 70 kDa protein, heat shock 60 kDa protein, galectin-1, calreticulin, tropomyosin alpha-3 chain (TPM3), tropomyosin alpha-4 chain (TPM4), perilipin-3, calumenin, and vimentin) were demonstrated on 2D gel with their densitometric analysis in Figure 6. The detailed information of the identified 9 proteins is presented in Supplementary Table.

Various types of stress are known to induce HSP expressions in cells. Many physiological processes including protein folding, transport, and assembly are mainly related with these proteins [24]. Consistently, our proteomics data showed that HSP70 and HSP60 expressions increased in senescent cells compared to young cells in control conditions. It has been shown that the expression levels of certain chaperones of the HSP70 family (HSPA1A/B and HSPA2) increased with age in fibroblast cells. This increase in HSP70 family can be explained by the response of the senescent fibroblasts to increasing amount of unfolded proteins due to the decrease in proteasome activity [25].

Galectin-1 serves as a potential regulator for cell adhesion, migration, and growth through protein/glycan and protein/protein interactions. Endoplasmic reticulum chaperone calreticulin has an important role on partially folding glycoproteins to be degraded by proteasome. Calreticulin acts as a phagocytosis facilitator in apoptotic cells [26]. In a two-dimensional gel-based proteomic study of fibroblast cells, an increase in galectin-1 and calreticulin protein expressions was observed with aging. Since calreticulin is essential for intracellular calcium homeostasis, deletion of calreticulin gene affects quality control mechanism in the endoplasmic reticulum and activates the unfolded protein response [27]. As shown in Figure 6, while galectin-1 expression decreased with senescence, an increase in calreticulin levels was observed. In addition, calreticulin expression was suppressed by mild hyperthermia and increased by a recovery process after hyperthermia conditions. TPM3 expression increased in young cells in mild hyperthermia conditions. On the other hand, while TPM3 expression increased in senescent cells, it decreased with hyperthermia in the same cells. TPM4 expression decreased in senescent cells. In the hyperthermia condition, TPM4 expression was increased in both young and senescent cells. In addition, our study demonstrated that the expression levels of vimentin decreased in both young and senescent cells due to mild hyperthermia and increased following the recovery of hyperthermia conditions.

## 4. Conclusions

Our results showed that the rapid increase in HSP40/70 levels may be the main protection response against mild hyperthermia both in young and senescent cells. On the other hand, cellular stresses induced either by hyperthermia or replicative senescence resulted in the impairment of the proteasomal activity. However, when HSP70 expression was inhibited, the proteasome activity was found to increase to maintain protein homeostasis. Collectively, our data suggest the involvement of HSP70 and the proteasome activity, as well as their connection, in stress conditions including senescence and mild hyperthermia.

## Data Availability

The data used to support the findings of this study are available from the corresponding author upon request.

## Supplementary Materials

Supplementary Materials Supplementary Figure 1: effects of mild hyperthermia and recovery on the viability of young and senescent fibroblast cells. Supplementary Figure 2: protein turnover in young and senescent fibroblast cells. Supplementary Figure 3: effect of mild hyperthermia and recovery on protein turnover in young fibroblast cells. Supplementary Figure 4: HSP70 silencing and protein turnover in young fibroblast cells. Supplementary Table: detailed information of nine proteins identified by MALDI-TOF MS in young and senescent fibroblasts.
