# Supplementary material for: HSP70 Inhibition Leads to the Activation of Proteasomal System under Mild Hyperthermia Conditions in Young and Senescent Fibroblasts
Source: Oxid Med Cell Longev. 2020 Feb 27;2020:9369524. doi: 10.1155/2020/9369524 (PMC7064868; doi:10.1155/2020/9369524)
Supplement: Supplementary Materials — Supplementary Figure 1: effects of mild hyperthermia and recovery on the viability of young and senescent fibroblast cells. Supplementary Figure 2: protein turnover in young and senescent fibroblast cells. Supplementary Figure 3: effect of mild hyperthermia and recovery on protein turnover in young fibroblast cells. Supplementary Figure 4: HSP70 silencing and protein turnover in young fibroblast cells. Supplementary Table: detailed information of nine proteins identified by MALDI-TOF MS in young and senescent fibroblasts. [file 9369524.f1.doc]

**Supplementary methods**

**Cell Viability**

Cell viability was determined by MTT assay (Sigma-Aldrich, USA). 2 x10^5^ fibroblast cells were seeded. When the cells were confluent, 10 μl of the MTT labeling reagent (0.5 mg/ml) was added to cells and incubated for 4 h in a humidified atmosphere of 37°C, 5% CO_2_. Then, 100 μl of the solubilization solution added into each well. After dissolving formazan crystals, the absorbance of the samples was measured at 595 nm using a plate reader. The optical density of each well was determined in order to quantify cell viability.

**Determination of protein turnover in young and senescent cells**

Fibroblast cells were cultivated until they reached 5x10^6^ cells/ml. Cells were incubated with [^35^S] methionine/cysteine at the end activity of 100 μCi/ml in methionine and cysteine-free minimal essential Eagle's medium at 37 °C for 16 h. For proteolysis measurement, equal volume of 20% trichloroacetic acid were added to the cell supernatant and incubated for 2 h at 4°C and centrifuged at 14,000 g for 10 min at 4°C.  Scintillation counting was performed with the acid-soluble supernatant after centrifugation at 14,000 × g for 15 min at 4 °C. The acid-soluble counts were calculated as (acid soluble sample counts/incorporated counts) × 100. For protein aggregate measurement, detergent solubility was taken as a measure. After collecting the medium for proteolysis measurement, cells were scraped, and pellets were resuspended in a detergent solution consisting of 1% Triton X-100, 0.5% sodium deoxycholate, and 0.1% SDS in 10 mm Tris-HCl, 1 mm EDTA (pH 8). After cell lysis at 4 °C for 15 min, samples were centrifuged at 13,000 × g for 10 min. Supernatants were counted as detergent-soluble proteins, and pellets were dissolved in 1 n NaOH and counted as detergent-insoluble proteins.

**Supplementary figures**


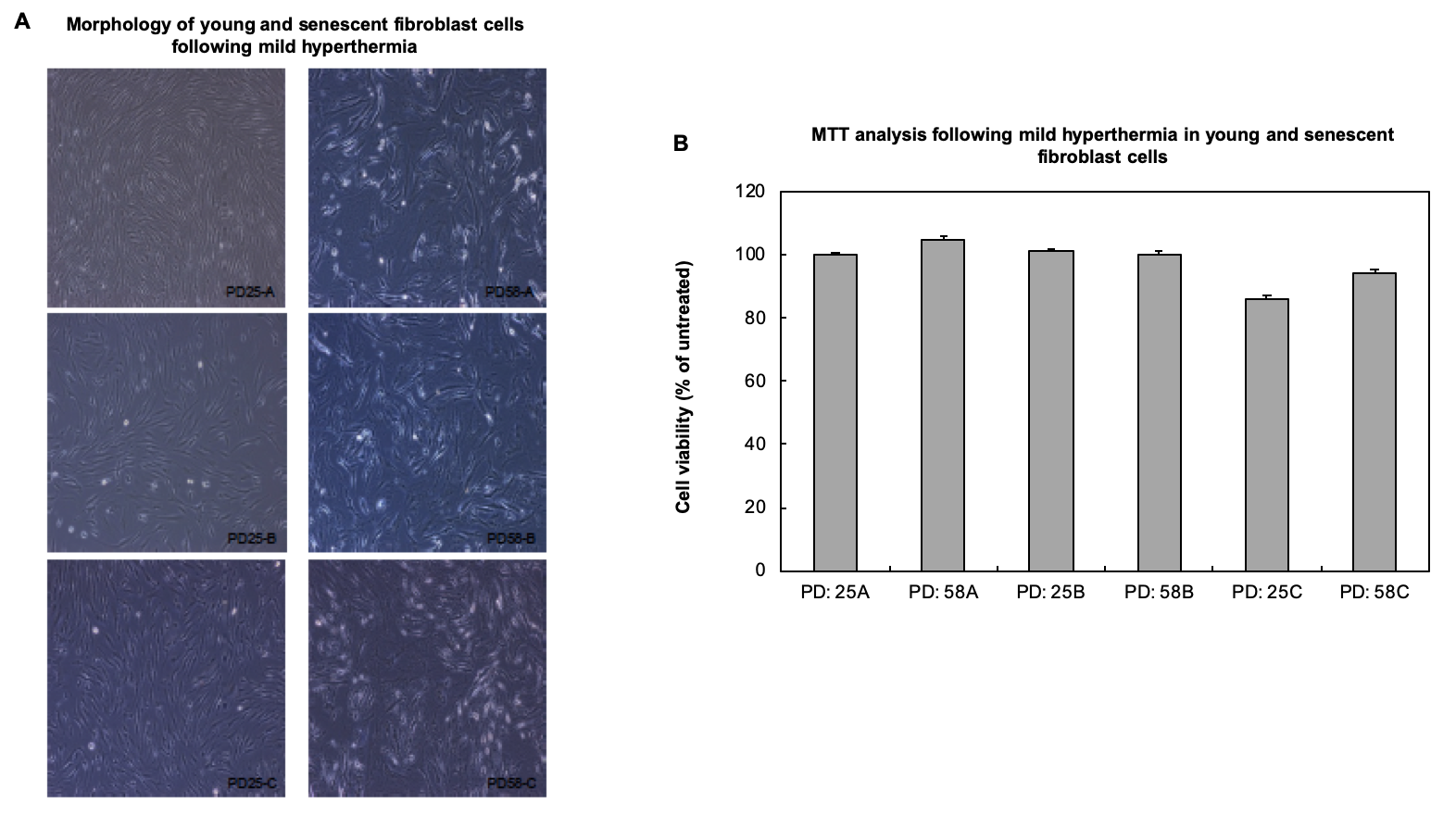


**Supplementary Figure 1. Effects of mild hyperthermia and recovery on the viability of young and senescent fibroblast cells**

Cells were treated with mild hyperthermia at 42 °C for 1 h and used immediately for analysis or cultured at 37 °C for 3 h. General morphology and cell viability were analyzed by light microscopy **(A)** and MTT assay **(B)** respectively.

Data are expressed as mean ± S.D. p> 0.05 in all groups (n=3).


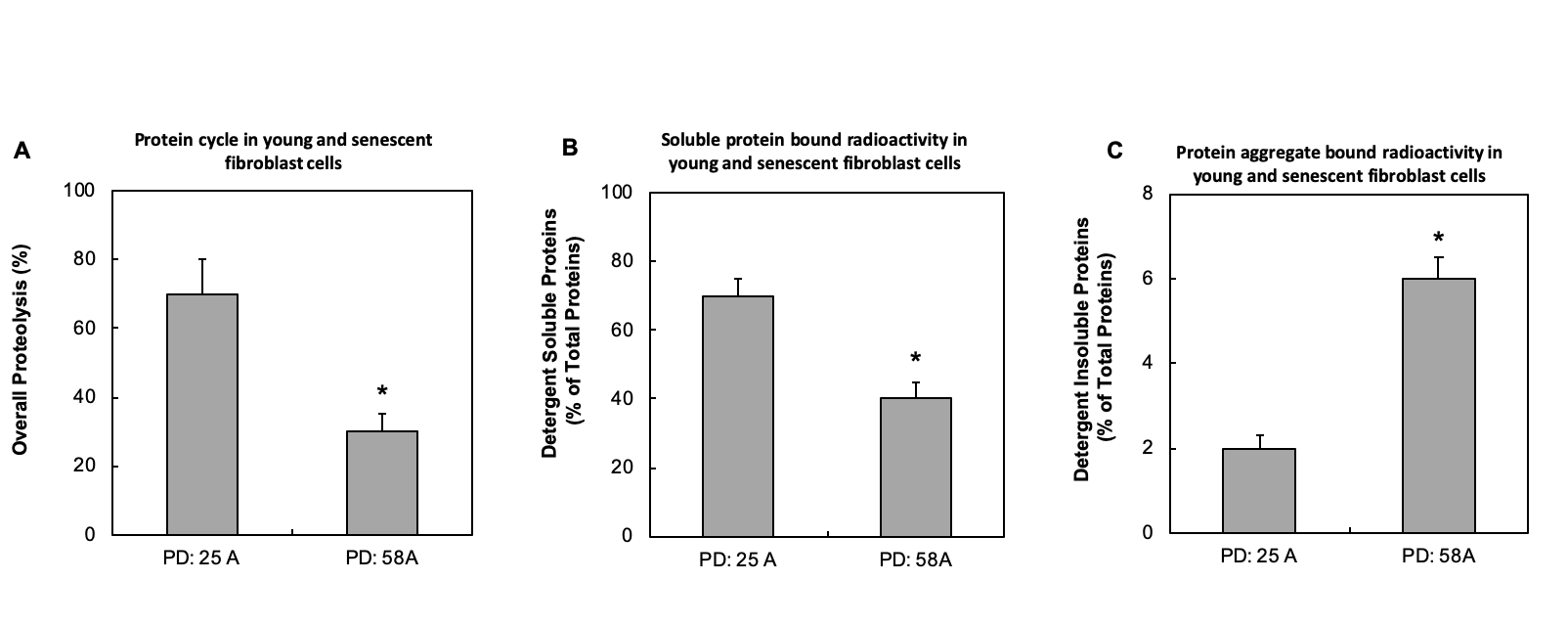


**Supplementary Figure 2. Protein turnover in young and senescent fibroblast cells**

The effect of senescence on the overall protein degradation in fibroblasts was tested. Endogenous proteins in fibroblasts were metabolically radio-labeled with [^35^S] Met/Cys and percent degradation **(A)**, detergent-soluble **(B)** and detergent insoluble **(C)** of [^35^S] cellular proteins was measured liquid scintillation counting.

Data are expressed as mean ± S.D. *p < 0.05 vs. PD: 25A.


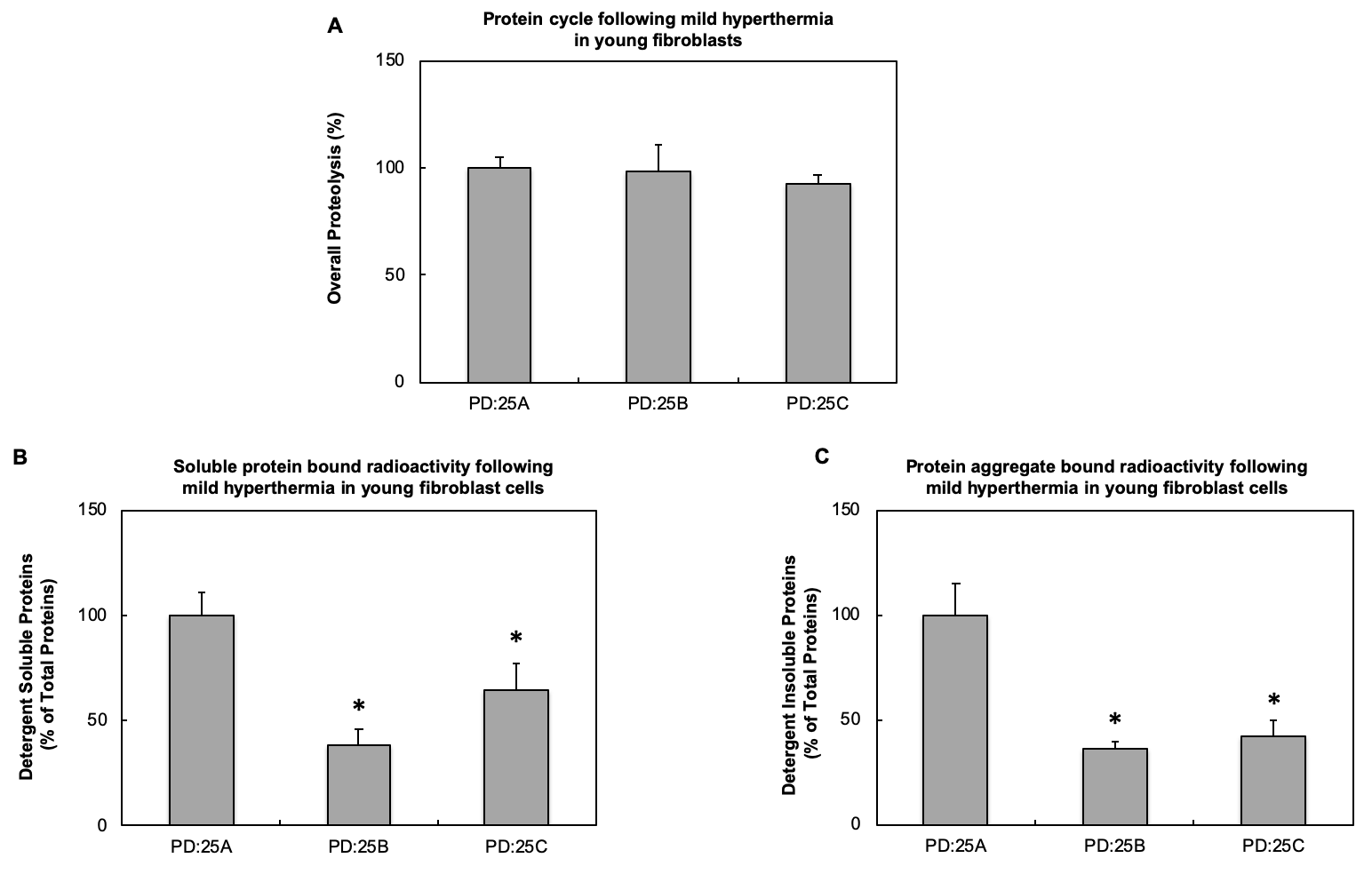


**Supplementary Figure 3. Effect of mild hyperthermia and recovery on protein turnover in young fibroblast cells**

Cells were treated with mild hyperthermia at 42 °C for 1 h and used immediately for analysis or cultured at 37 °C for 3 h. Endogenous proteins in young fibroblasts were metabolically radio-labeled with [^35^S] Met/Cys and percent degradation **(A)**, detergent-solubility **(B)** and detergent insolubility **(C)** of [^35^S] cellular proteins was measured by liquid scintillation counting.

Data are expressed as mean ± S.D. *p < 0.05 vs. PD: 25A.


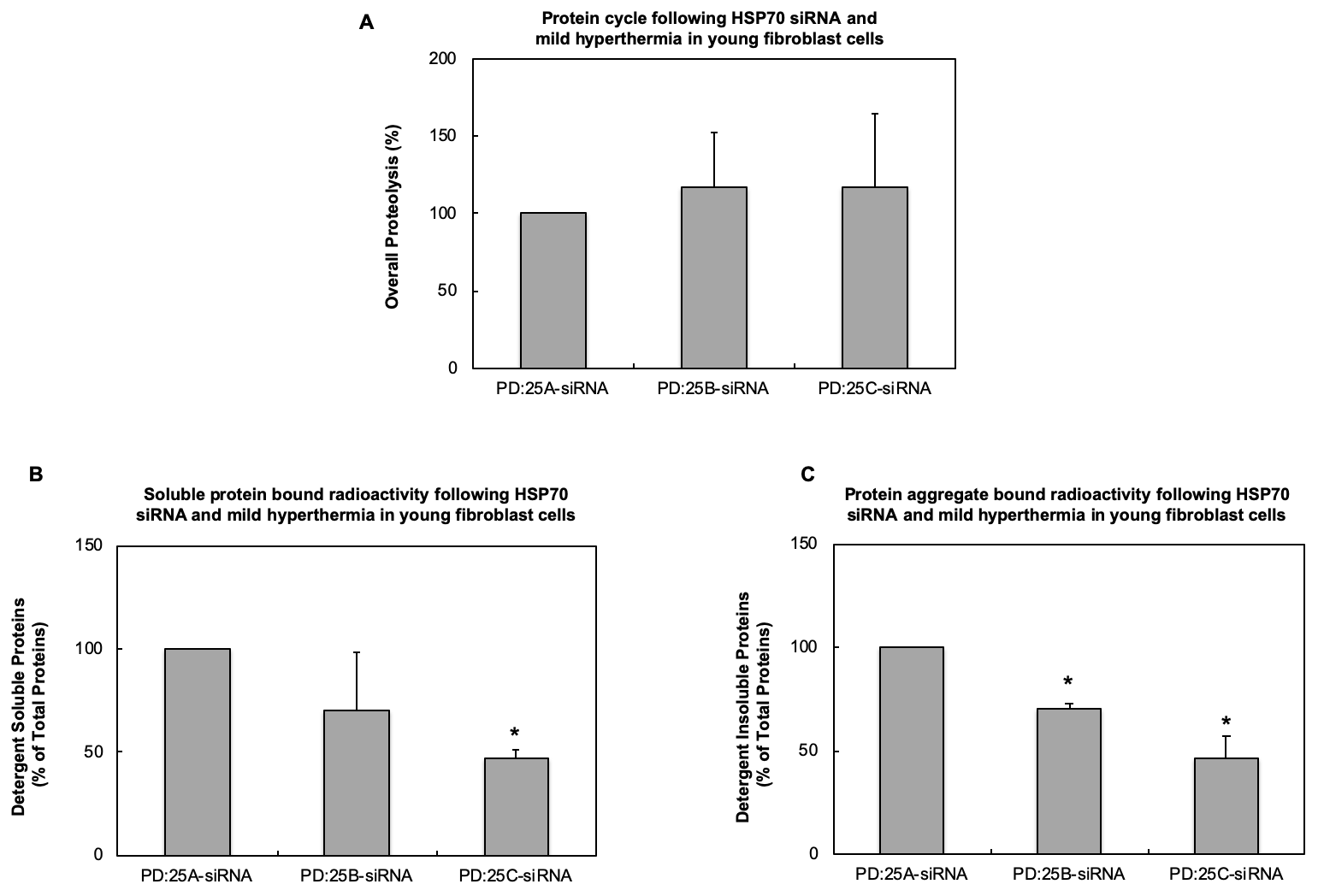


**Supplementary Figure 4. HSP70 silencing and protein turnover in young fibroblast cells**

Cells Cells were transfected with HSP70 and were treated with mild hyperthermia at 42 °C for 1 h and used immediately for analysis or cultured at 37 °C for 3 h. Endogenous proteins in young fibroblasts were metabolically radio-labeled with [^35^S] Met/Cys and percent degradation **(A)**, detergent-solubility **(B)** and detergent insolubility **(C)** of [^35^S] cellular proteins was measured by liquid scintillation counting.

Data are expressed as mean ± S.D. *p < 0.05 vs. PD: 25A.

**Supplementary Table:** Detailed information of nine proteins identified by MALDI-TOF MS in young and senescent fibroblasts.

| **Accession number** | **Protein name** | **Score** | **pI** | **Mw (kDa)** |
| --- | --- | --- | --- | --- |
| P11142 | Heat shock 70 kDa protein | 69 | 5,61 | 70,9 |
| P10809 | Heat shock 60 kDa protein | 95 | 5,24 | 61 |
| P09382 | Galectin-1 | 137 | 5.30 | 14 |
| P27797 | Calreticulin | 151 | 4,29 | 48 |
| P06753-2 | Tropomyosin alpha-3 chain | 219 | 4,75 | 29 |
| P67936 | Tropomyosin alpha-4 chain | 235 | 4,69 | 28,5 |
| O60664 | Perilipin-3 | 122 | 5,30 | 47 |
| O43852 | Calumenin | 56 | 4,46 | 37,1 |
| P08670 | Vimentin | 61 | 5,05 | 53,6 |
